# Novel pancreatoscope improves diagnostic yield of visual findings for intraductal papillary mucinous neoplasms

**DOI:** 10.1055/a-2197-8949

**Published:** 2023-11-14

**Authors:** Takeshi Ogura, Taro Iwatsubo, Kimi Bessho, Nobuhiro Hattori, Hiroki Nishikawa

**Affiliations:** 1 Endoscopy Center, Osaka Medical and Pharmaceutical University Hospital, Osaka, Japan; 2 2nd Department of Internal Medicine, Osaka Medical and Pharmaceutical University, Osaka, Japan

Intraductal papillary mucinous neoplasms (IPMNs) are characterized by mucin-producing neoplastic epithelium, and, as a result, pancreatic dilatation is observed 1 . Among IPMNs, main-duct IPMN (MD-IPMN) has high potential for malignancy, and surgical resection is recommended 2 . Preoperative diagnosis at the site of the mural nodule has clinical impact, allowing the necessary surgical margins to be determined. According to a previous study 3 , pancreatoscopy has a higher detection rate than other diagnostic modalities. A single-operator pancreatoscope may be useful 4 , but because the working channel is small, aspirating mucin may sometimes be a challenge, and therefore the diagnostic yield may be reduced because of poor visualization. Recently, a novel pancreatoscope has become available (eyeMax; Micro-Tech Co., Ltd., Nanjing, China) ( Fig. 1 ). The diameter of its working channel is 1.8 mm, and the dedicated biopsy forceps, with a cup length of 1.6 mm, allows large amounts of histological tissue to be obtained. Successful preoperative diagnosis for determination of the surgical margins of MD-IPMN using this novel pancreatoscope is described.

**Fig. 1:**
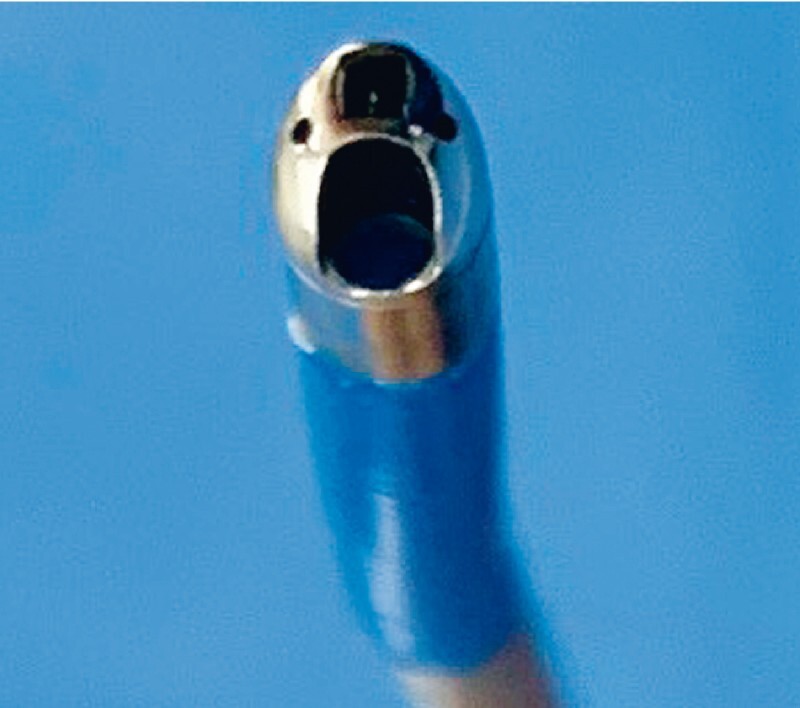
Novel pancreatoscope (eyeMax; Micro-Tech Co., Ltd., Nanjing, China). The diameter of the working channel is 1.8 mm.

A 72-year-old man was admitted to our hospital with a MD-IPMN. No mural nodule could be detected on computed tomography or endoscopic ultrasonography. Therefore, pancreatoscopic evaluation was attempted. First, the duodenoscope was inserted, and mucin was observed flowing from the ampulla of Vater ( Fig. 2 ). Then, the novel pancreatoscope was inserted within the pancreatic duct. Because of the massive amount of mucin present, the findings on pancreatoscopy were initially poor ( Fig. 3 ). However, because the novel pancreatoscope has a large working channel, the mucin was easily aspirated ( Fig. 4 ), allowing a mural nodule to be detected at the body of the pancreas ( Fig. 5 ). Finally, forceps biopsy under pancreatoscopic guidance was performed ( Video 1 ). On histological examination, adenocarcinoma was diagnosed, and distal pancreatectomy was performed successfully.

**Fig. 2:**
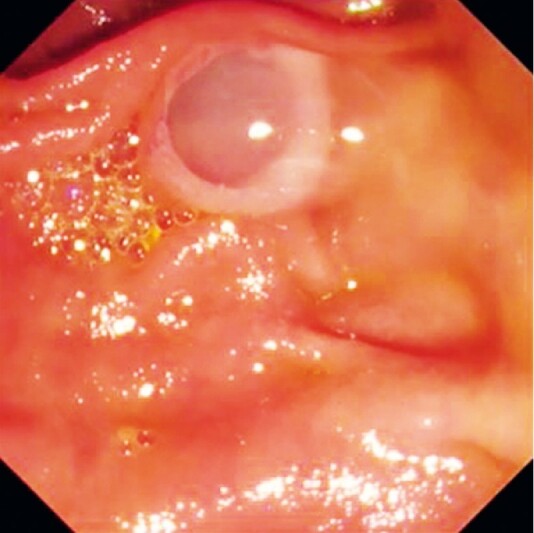
Mucin is observed flowing from the ampulla of Vater.

**Fig. 3:**
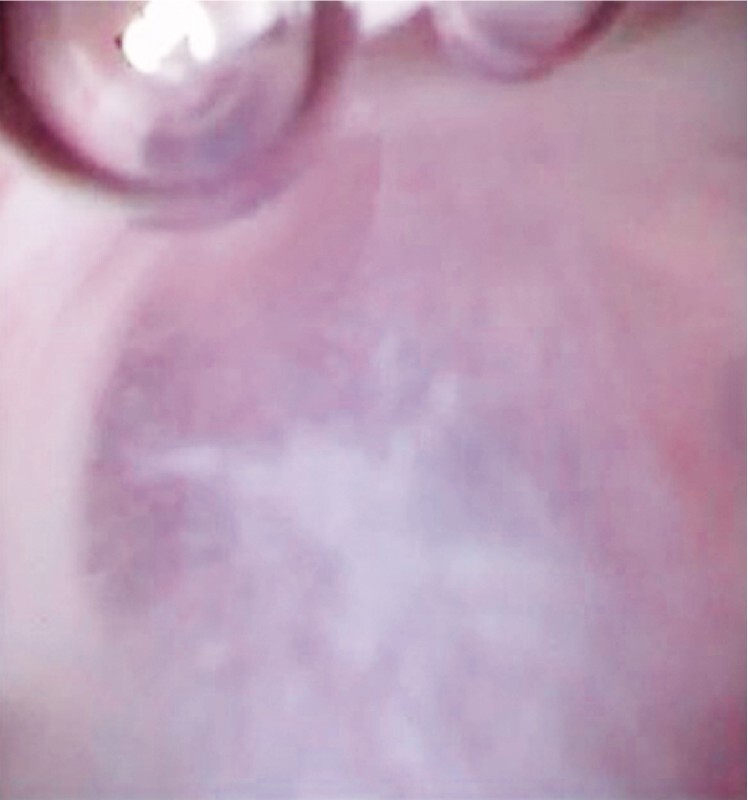
The pancreatoscopic view is obscured by mucin.

**Fig. 4:**
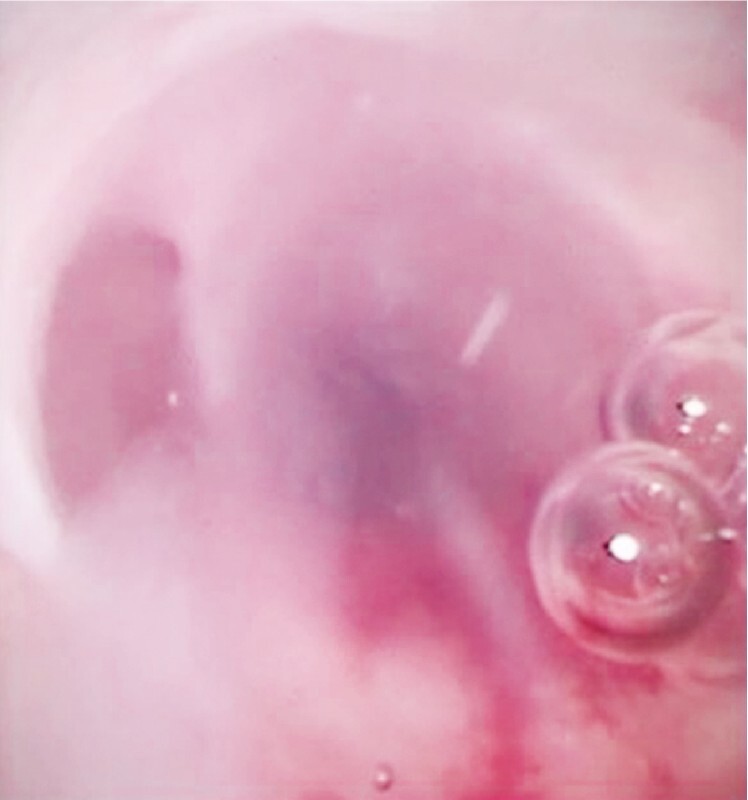
Mucin is easily aspirated.

**Fig. 5:**
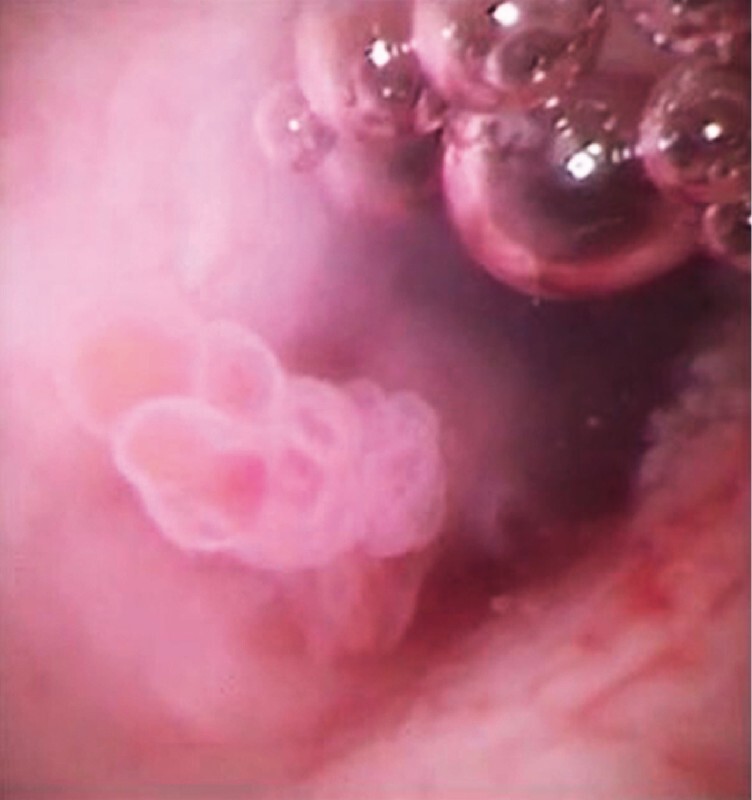
A mural nodule can now be clearly observed.

**Video 1** Diagnostic pancreatoscopy in a patient with a main-duct intraductal papillary mucinous neoplasm. The pancreatoscope is inserted. The view is obscured by mucin, but the mucin is easily aspirated. A mural nodule is then clearly observed. Forceps biopsy is successfully performed.


In conclusion, this novel pancreatoscope may be useful in determining surgical margins in cases of MD-IPMN.

Endoscopy_UCTN_Code_CCL_1AZ_2AB

## Notes

Endoscopy E-Videos

https://eref.thieme.de/e-videos
*E-Videos* is an open access online section of the journal *Endoscopy* , reporting on interesting cases and new techniques in gastroenterological endoscopy. All papers include a high-quality video and are published with a Creative Commons CC-BY license. Endoscopy E-Videos qualify for HINARI discounts and waivers and eligibility is automatically checked during the submission process. We grant 100% waivers to articles whose corresponding authors are based in Group A countries and 50% waivers to those who are based in Group B countries as classified by Research4Life (see: https://www.research4life.org/access/eligibility/ ). This section has its own submission website at https://mc.manuscriptcentral.com/e-videos
